# Exploring nurses’ experiences of providing spiritual care to cancer patients: a qualitative study

**DOI:** 10.1186/s12912-024-01830-2

**Published:** 2024-03-27

**Authors:** Huda A. Anshasi, Mirna Fawaz, Yousef M. Aljawarneh, Ja’far M. Alkhawaldeh

**Affiliations:** 1 Department of Nursing, College of Health Sciences, University of Fujairah, Fujairah, United Arab Emirates; 2 College of Health Sciences, American University of the Middle East, Egaila, Kuwait; 3 The School of Nursing, Higher Colleges of Technology, Abu Dhabi, P.O. Box: 1626, United Arab Emirates; 4 Faculty of Nursing, Isra University, Isra University Office 11622, Amman, P.O. Box 33 and 22, Jordan

**Keywords:** Spirituality, Spiritual care, Nurses’ experiences, Patients with cancer, Qualitative study

## Abstract

**Purpose:**

This study aims to explore nurses’ experiences of delivering spiritual care in an oncology setting.

**Methods:**

An exploratory- descriptive qualitative design. Focus groups were conducted to gather data. The discussions were recorded and transcribed verbatim to ensure accuracy, credibility, and reliability. Inductive thematic analysis was performed to analyze the narratives, and the study’s reporting followed the Consolidated criteria for reporting qualitative studies.

**Results:**

The study revealed four major themes: the spirituality from the perspective of oncology nurses, recognition of patients’ spiritual needs, delivery of spiritual care to patients with cancer, and barriers in implementing spiritual care. The participants identified insufficient staff, lack of time, and insufficient knowledge and skills as obstacles to delivering spiritual care.

**Conclusion:**

This study offers a thorough understanding of how nurses provide spiritual care in an oncology setting. It is recommended that organizational interventions, such as increasing staff numbers, be implemented to enable nurses to provide more compassionate care. Furthermore, nursing curriculums should incorporate purposeful engagement and focused debriefing related to spiritual care to better equip nurses in identifying and meeting the spiritual needs of their patients.

**Supplementary Information:**

The online version contains supplementary material available at 10.1186/s12912-024-01830-2.

## Introduction

Patients with cancer often face a wide range of spiritual concerns and needs, from initial diagnosis to treatment, survival, recurrence, and dying [1, 2]. Research has consistently shown that cancer patients with greater levels of spiritual well-being experience lower levels of anxiety and depression, in addition to an improved quality of life [3–5]. This highlights the essential of offering spiritual care to cancer patients to effectively meet their spiritual needs and improve their well-being.

Recently, there has been an increasing interest in exploring the spiritual needs of cancer patients [6–8]. Previous studies have emphasized the significance of addressing spiritual needs while considering cultural differences in order to provide holistic, patient-centered care [9–11]. Therefore, healthcare professionals must recognize and adapt culturally appropriate and patient-centered care to meet the spiritual requirements of cancer patients.

Nurses play a crucial role in assessing the patients’ spiritual requirements and delivering spiritual care due to their close relationship with patients and frequent interaction with them [12, 13]. Nurses’ perceptions of spiritual care significantly influence the way they deliver spiritual nursing care [14, 15].

In Lebanon, spiritual care is becoming increasingly recognized for its importance within the healthcare sector [16]. However, there is a paucity of empirical evidence regarding how nurses deliver this type of care to cancer patients in the country. Gaining insight into the experiences of nurses will enable more effective development of spiritual care in Arabic nations, particularly Lebanon.

## Background

Spirituality is increasingly being recognized as a fundamental element of person-centered care and a pivotal factor in how cancer patients cope with their illness [1, 17]. In line with Florence Nightingale’s philosophy of care, spirituality is inherent in human beings, and is the most profound and powerful source of healing [18]. Thus, nurses have an important responsibility to attend to spiritual dimensions of care and create a healing atmosphere for patients.

Spirituality and spiritual care are concepts affected by a nurse’s ethnicity, religion, education level, and clinical experience [19–21]. Nursing specialties have varying perceptions; research shows that mental health care and palliative care nurses have better understanding and skills in providing spiritual care than others [22, 23]. Several authors recommend conducting more studies on nursing spiritual care in different settings to gain a better understanding of this phenomenon [15, 24–26].

The way in which nurses view and comprehend spirituality and spiritual care can impact their ability to provide it to their patients [20, 22]. However, some scholars suggest that these perspectives are rooted in Western culture and may not reflect the beliefs of nurses from other cultures [24, 27, 28]. Despite this, there has been no study conducted on how nurses in Arabic countries, particularly in Lebanon, deliver spiritual care to cancer patients. Therefore, this study aims to explore nurses’ experiences of delivering spiritual care in an oncology setting in Lebanon.

The key objectives of this study were to (1) define the concept of spirituality based on the perspective of Lebanese nurses working in oncology, (2) assess the current spiritual care practices, and (3) explore the barriers in delivering spiritual care. The study’s findings offer a valuable understanding of the experiences of Lebanese nurses in providing spiritual care. These findings can serve as a resource for nurses, healthcare providers, and nursing educators to enhance the current situation and plan for future improvements.

## Methods

### Design

An exploratory descriptive qualitative design was employed to understand the experiences and viewpoints of nurses regarding spirituality and spiritual care in Lebanon. This design was selected as it allowed participants to freely share their experiences and enabled the researcher to investigate emerging themes. The study followed the Consolidated criteria for reporting qualitative studies [29].

### Setting and participants

A purposive sample of 20 Lebanese nurses was recruited from one of the major hospitals in the Bekaa Valley, Lebanon. Registered nurses with at least six months of work experience in oncology settings who were willing to participate in the study were included in this study. To prevent bias related to language differences and cultural distinctions that may affect the presentation of spirituality within the context of Lebanon, international nurses were excluded. Every nurse that was approached consented to an interview.

### Data collection

Data was collected through five semi-structured, face-to-face focus groups. Each group consisted of four participants to ensure that everyone had the chance to express themselves freely. The sessions lasted 30–60 min and were conducted in private offices to ensure privacy and minimize interruptions. The facilitator (second author) and moderator (first author) were not employed by the selected hospital to avoid bias and coercion. They explained the focus group’s work method, used open-ended questions, and probed until a full understanding was achieved.

All five focus groups were asked the same questions, including defining spirituality and providing spiritual care, as well as exploring barriers and strategies in delivering spiritual care in the oncology context. Refer to for more information.

Supplementary questions were used to clarify all information, and the facilitator ensured that the conversation flowed smoothly. The moderator observed and took field notes, and all discussions were audiotaped and transcribed verbatim for accuracy, credibility, and reliability purposes. The findings did not reveal any new information after the twenty participants.

### Data analysis

The study employed an inductive thematic analysis method to analyze the qualitative narratives [30]. The authors read the transcripts, coded the text, created categories, and identified themes. The three authors independently conducted the data analysis, and the results were later discussed and modified until a consensus was reached. To fully understand the participants’ perspective, the authors read the transcripts multiple times.

The transcripts were coded by analyzing each line and assigning a word or phrase that captured the data’s essence. Some codes, like “unique”, “the essence of living”, and “whole”, were grouped under the spirituality as perceived by nurses. The codes were refined and regrouped to form themes that represented meaningful units on experiences towards providing spiritual care by oncology nurses. The themes were carefully scrutinized to ensure that the data were accurately represented. Disagreements were settled by conducting a secondary analysis of the interviews until the three authors achieved a shared coding agreement.

### Trustworthiness of the study

To achieve rigor, the interviews were conducted by a single interviewer, who engaged with the participants for a prolonged period to ensure authenticity. The interviews continued until saturation was reached, meaning that no new elements were obtained from the analysis of the interviews. Concurrent analysis was also employed to probe emerging themes in sequential interviews. For consistency, the same interview guide was used in all focus groups. The authors reviewed the themes generated and ensured no data was left out. Direct quotes were also used to support the study’s findings, giving participants a voice.

Moreover, data validation was performed by qualified personnel through peer checking. Three authors analyzed the data independently and agreed on common themes through discussion and comparison. To establish credibility and confirmability, member-checking was utilized by returning themes and categories to the participants for confirmation of data interpretation.

Furthermore, to ensure the study’s findings were transferable, the authors utilized external member-checking. This involved three individuals with similar experiences of the study subject determining the congruence between their own experiences and the study’s findings.

To ensure the applicability of the study’s results to various settings, the research findings were shared with multiple nurses who were not part of the study. They were then requested to evaluate how closely the study’s outcomes aligned with their own experiences.

### Ethical considerations

The study protocol was approved by the IRB of Rayak University Hospital (IRB Study No. ECO-R-205). Participants were informed about the voluntary nature of their participation and the option to withdraw without consequences. They completed an informed consent from after learning the research objectives and procedures. Participants were also guaranteed anonymity and the confidentiality of their data would be strictly protected.

## Results

### Characteristics of participants

Results show that the study had 20 participants who met the criteria, with 60.0% (12) being female. The average age was 30.6 (SD = 2.9) and ages ranged from 24 to 45 years. Nursing experience also varied, with registered nurses in oncology settings having work experience ranging from one to six years, and overall nursing experience ranging from three to nine years. It is noteworthy that all the nurses who participated in this study were of the Muslim faith.

### Nurses’ experiences in spiritual care in oncology setting

Four main themes emerged from the analysis that were present in all three groups: “Spirituality from the Perspective of Oncology Nurses”, “Provision of Spiritual Care to Cancer Patients”, “Recognition of Patients’ Spiritual Needs”, and “Barriers in implementing Spiritual Care”. The codes utilized in each of these main themes are outlined in Table 1.

**Table 1:** Four major themes that emerged from codes

Themes	Codes
Spirituality from the Perspective of Oncology Nurses	The essence of living Unique Whole Universal Within the context of religion
Recognition of patients’ spiritual needs	Communication Observation of the patient’s surrounding Expression of feelings Health status or diagnosis of patients
Provision of spiritual care to cancer patients	Facilitating prayers. Encouraging the patients to read and/ or listen to Quran. Encouraging the patients’ families to participate in the spiritual care. Encouraging the patients to trust Allah (God).
Barriers in implementing spiritual care	Insufficient staff Lack of time Insufficient knowledge and skills

#### The spirituality from the perspective of oncology nurses

The first theme that arose centered on the conceptualization of spirituality as described by the oncology nurses who participated. They conveyed that spirituality is not solely linked to religion; instead, it is viewed as a personal and subjective experience, although the majority directly associated it with religious belief.

The codes “within the context of religion”, “the essence of living”, “unique”, “whole”, and “universal” were merged to become the theme “spirituality from the perspective of oncology nurses”.

A majority of nurses defined spirituality within the context of religion. For instance, one of the nurses stated: “*Spirituality is believing in Allah (God)*” (Male nurse, focus group 2).

Furthermore, many nurses described spirituality as the essence of their living. One of the nurses stated: *“It means the essence of my being”*. (Female nurse, focus group 1)

Another nurse said: *“In my view it is what makes me feel what makes me. It is the essence of living”* (Female nurse, focus group 2).

For some of the nurses, spirituality was about what makes them unique, individual and whole. For instance, one of the nurses stated: “*Spirituality is a component of everything… whole mind, body, and emotional… all gathered into one”* (Male nurse, focus group 1). Another nurse said: *“It makes somebody feel whole”* (Female nurse, focus group 2).

Another nurse said: *“In my view, spirituality is different to every person. For some people, it is the religious beliefs in their lives, whereas other people it is not necessarily about their religious”* (Male nurse, focus group 3).

Other nurses described Spirituality as universal. For example, one of the nurses stated: *“It is difficult to define the spiritual part of a person, but we all have it whether we recognize that or not. Spirituality is universal”* (Female nurse, focus group 3).

#### Recognition of patients’ spiritual needs

Observation of the patient’s surrounding, expression of feelings, health status or diagnosis, and communication were among the codes that shaped the theme of recognizing patients’ spiritual needs. According to many nurses, paying attention to the patient’s surroundings and being receptive to their emotional expressions helped them identify the patient’s spiritual needs. For instance, one of the nurses stated: *“Yesterday, I entered my patient’s room to find her in tears. I sat down next to her and held her hand, providing her with a tissue. In these moments, it is essential to establish a connection with your patient on a spiritual level, allowing them to express their feelings freely and openly.”* (Female nurse focus group 4).

Moreover, most of the nurses considered listening and communication will help gain an understanding of spiritual needs. For instance, one of the nurses stated: “*I think listening and communication will help nurse to assess patients’ needs”* (Male nurse focus group 1).

According to some nurses, patients who have just been diagnosed with cancer or are undergoing surgeries like mastectomy require spiritual care. For instance, a nurse stated: *“If somebody is newly diagnosed with cancer, people tend to doubt; they ask why this is happening to them. It is important to be able to connect with your patient on spiritual level… to basically allow them to voice their concerns, their belief.”* (Female nurse focus group 5).

#### Provision of spiritual care to cancer patients

The approach of offering spiritual care in clinical was explained by the participants. From these data, four ways that oncology nurses provide spiritual care through presence in their practice were identified: (1) facilitating prayers, (2) encouraging patients to read and/ or listen to Quran, (3) encouraging patients’ families to participate in spiritual care, and (4) encouraging patients to trust Allah (God).

The majority of nurses participating in this study conveyed that prayer was the prevailing approach for delivering spiritual care. For instance, one of the nurses stated: “*I really believe that prayer helps patient to feel better. So, I facilitate prayers for my patients by giving a reminder for daily prayers and finding space and time for praying”* (Male nurse focus group 2).

Furthermore, Family involvement in the care process is encouraged by the participants, who facilitate opportunities for them to pray and practice religious rituals with the patient. One of the nurses stated: *“I usually encourage the family members to participate in religious rituals with patient, such as offering them Zamzam water. Muslims believe that drinking Zamzam water can bring wellness to those who are sick.”* (Male nurse focus group 1).

Moreover, most of the nurses believe that reading the Quran can produce peace of mind and tranquility of the soul. Therefore, they encourage their patients to read and/ or listen to the Quran. For instance, one of the nurses stated: “*Cancer affects patient not only physically, but also psychologically. The Quran has the ability to heal the heart. By motivating patients to read or listen to the Quran, they can receive healing energy directly from Allah (God) to cure their illnesses.”* (Female nurse focus group 5).

Other nurses expressed that encouraging the patients to trust Allah (God) was a way for providing spiritual care. For instance, one of the nurses stated: *“I always encourage my patients to trust Allah (God) and say to them, Allah (God) will not leave you alone in whatever, and he will take care of all these things”* (Female nurse focus group 1).

#### Barriers in implementing spiritual care

Insufficient staff, lack of time, and insufficient knowledge and skills in providing spiritual care were identified as codes contributing to this theme. Participants considered that spiritual care is essential for cancer patients. However, lack of time and availability due to work overload were perceived as barriers to delivering spiritual care. As a result, nurses tended to prioritize the physical care needs of patients over their spiritual needs due to the organization of work based on functional positions of tasks and the prioritization of patient care. For instance, one of the nurses stated: *“I am usually focus on the disease, on the procedures that I have to do so, sometimes I do not have time to sit and listen to my patients”* (Male nurse focus group1).

Other nurse said: *“we are opening more and more beds, so the management need to have the appropriate staff-patient ratio.”* (Male nurse focus group 2).

Moreover, there were nurses who express concern about their lack of knowledge and skill in delivering spiritual care. For instance, one of the nurses stated: *“I didn’t really get any official education on spiritual care.”* (Female nurse focus group 2).

## Discussion

The study offered detailed depictions of nurses’ encounters with providing spiritual care in an oncology setting. As no previous study has investigated the nurses’ experiences of offering spiritual care to cancer patients in Lebanon, this study provides a distinct outlook on this domain.

The study has shown that most of the participants defined spirituality within the context of religion, especially in the Islamic context where religious practices and thoughts were seen as essential for spiritual growth and salvation [31].

This finding is in line with similar studies that revealed that nurses tend to associate spirituality with religion [19, 32]. However, it contradicts other studies that found that nurses described spirituality as being separate to a belief in religion [33, 34]. The finding’s consistency with certain studies and inconsistency with others may be attributed to spirituality encompassing culture, personal growth, experiences, and perspectives of life [35–37]. Moreover, this study discovered that the identification of patients’ spiritual needs could be achieved through communication, observation of their environment, and understanding their feelings. These techniques were similar to ones mentioned in prior research [38, 39].

One of the most important findings of the study was that spiritual nursing care interventions reported by the participants included religious-based interventions. These interventions involved facilitating prayers, suggesting that patients read and/ or listen to Quran, encouraging patients’ families to participate in spiritual care, and encouraging patients to trust Allah (God). These results align with prior studies on spiritual nursing care interventions which also incorporated religious-based interventions [40–42]. Previous studies have indicated that praying can enhance the sense of hope during critical times [43, 44]. Nurses have reported supporting patients by advising them to read and/or listen to the Holy Quran, in line with previous studies that have shown Holy Scriptures to provide spiritual comfort and reassurance to patients [45, 46]. However, the finding of this study indicates some of the nurses may have considered that encouraging the patients to trust Allah (God) is a way for providing spiritual care, while in fact it may be imposing their own beliefs.

Another important finding of the study was that spiritual care was deemed necessary by participants for cancer patients, but was rarely included in their daily activities. The primary reasons for this were related to work dynamics in oncology settings, with physical needs taking priority, and a lack of time and availability due to work overload. This aligns with previous research that found that the lack of time was the most important predictors of the lack of spiritual care [47, 48]. To address this issue, organizational-level interventions such as increasing staff could be implemented to allow for more time to provide compassionate and empathetic care, enabling nurses to identify and attend to the spiritual needs of patients. In addition, nurses should have a good understanding of the spiritual aspects of care, which are just as important as physical needs.

Concerns were expressed by certain nurses regarding inadequate knowledge and skills in the delivery of spiritual care. This discovery highlights the necessity of improving nurses’ abilities and knowledge in order to assist them in addressing patients’ spiritual requirements. Thus, nurse educators must assist nurses who work with cancer patients by establishing an ongoing educational program that focuses on spiritual nursing care. Furthermore, incorporating spirituality and spiritual care into the nursing curriculum is crucial, as highlighted by this discovery.

In term of limitations to this study, it involved oncology nurses only. Therefore, caution must be taken when extending the findings to other health professionals or nurses. Despite this, the personal experiences shared by each participant provide a distinct viewpoint on the subject. The study aimed to describe individual experiences and identify commonalities. While the results are transferable, they cannot be generalized. Future researchers should include nurses from diverse settings.

## Conclusion

The results of this qualitative study have provided a rich description of nurses’ experiences of delivering spiritual care in Lebanon context. Participants reported that they recognize patients’ spiritual needs by communicating, observing their environment, and expressing emotions. The majority of spiritual nursing care was rooted in religious beliefs and practices, thus highlighted the significance of religion in healthcare provision. Participants identified insufficient staff, lack of time, and insufficient knowledge and skills as barriers to providing spiritual care. To ensure time for compassion and empathy, interventions at an organizational level, such as increasing staff, should be implemented. In addition, nurse educators should provide continual education programs to support nurses working with cancer patients in relation to spiritual nursing practice. The results of this study are transferable, but not generalizable. Further research is necessary to examine nurses’ experiences of delivering spiritual care in different settings.

## Relevance to clinical practice

Awareness of spiritual aspects of care is equally important as the physical needs that nurses attend to. Nurse educators should support nurses working with cancer patients by developing a continuous education program related to spiritual nursing care practice. Furthermore, organizations can implement interventions to increase staff numbers and ensure that nurses have enough time to show compassion and empathy to their patients, enabling them to identify and provide spiritual care.

### Electronic supplementary material

Below is the link to the electronic supplementary material.

Supplementary Material 1

## Data Availability

Interview transcripts are available from the corresponding author on reasonable request.

## Declarations

### Declaration of AI and AI-assisted technologies in the writing process

The authors declare that no AI and AI-assisted technologies in the writing this manuscript.

## References

[1] Puchalski CM, King S, Ferrell B (2018). Spiritual considerations. Hematol Oncol Clin North Am.

[2] Wisesrith W, Sukcharoen P, Sripinkaew K (2021). Spiritual care needs of terminal Ill Cancer patients. Asian Pac J cancer Prevention: APJCP.

[3] Chen J (2021). Association between spiritual well-being, quality of life, anxiety and depression in patients with gynaecological cancer in China. Medicine.

[4] Cheng Q (2019). Improving spiritual well-being among cancer patients: implications for clinical care. Support Care Cancer.

[5] Zare A (2019). The relationship between spiritual well-being, mental health, and quality of life in cancer patients receiving chemotherapy. J Family Med Prim Care.

[6] Du S (2022). Spiritual needs and their associated psychosocial factors among women with breast cancer: a cross-sectional study. J Adv Nurs.

[7] Damen A (2022). Spiritual well-being and Associated Factors in Dutch patients with Advanced Cancer. J Pain Symptom Manag.

[8] Sastra L, et al. Spiritual needs and influencing factors of Indonesian muslims with Cancer during hospitalization. Jornal of transcultural nursing. Nursing; 2021. 10.1177/1043659620908926 32167014

[9] Devi M, Fong K (2019). Spiritual experiences of women with breast Cancer in Singapore: a qualitative study. Asia Pac J Oncol Nurs.

[10] Astrow A (2018). Spiritual needs and perception of quality of care and satisfaction with Care in Hematology/Medical Oncology patients: a multicultural Assessment. JPMS.

[11] López-Sierra HE, Rodríguez-Sánchez J (2015). The supportive roles of religion and spirituality in end-of-life and palliative care of patients with cancer in a culturally diverse context: a literature review. Curr Opin Support Palliat Care.

[12] Timmins F, Caldeira S (2017). Assessing the spiritual needs of patients. Nurs Stand.

[13] Zumstein-Shaha M, Ferrell B, Economou D. Nurses’ response to spiritual needs of cancer patients. Eur J Oncol Nurs. 2020; 48. 10.1016/j.ejon.2020.101792 32947158

[14] Galutira GD, Valenzuela JP, Basatan CJ, Castro-Palaganas E (2019). Spirituality and spiritual care in nursing: a literature review. Philipp J Nurs.

[15] Labrague L (2016). Filipino nurses’ spirituality and provision of spiritual nursing care. Clin Nurs Res.

[16] Abu-Saad Huijer H, Bejjani R, Fares S (2019). Quality of care, spirituality, relationships and finances in older adult palliative care patients in Lebanon. Annals Palliat Med.

[17] Fallahi L, Abdollahi F. Understanding the role of spirituality and faith in relation to Life Expectancy and End of Life Experience in Terminally-ILL Cancer patients. Gerontol Geriatric stud. 2017; 1(4).

[18] Macrae J (1995). Nightingale’s spiritual philosophy and its significance for modern nursing. Image: J Nurs Scholarsh.

[19] Han KH, Hung KC, Cheng YS, Chung W, Sun CK, Kao CC (2023). Factors affecting spiritual care competency of mental health nurses: a questionnaire-based cross-sectional study. BMC Nurs.

[20] Murgia C, Notarnicola I, Caruso R, De Maria M, Rocco G, Stievano A (2022). Spirituality and religious diversity in nursing: a scoping review. Healthc (Basel Switzerland).

[21] Hsieh S (2020). Factors associated with spiritual care competencies in Taiwan’s clinical nurses: a descriptive correlational study. J Clin Nurs.

[22] Meurs JV, Breedveld R, Geer JV, Leget C, Smeets W, Koorneef R, Vissers K, Engels Y, Wichmann A (2023). Role-perceptions of Dutch spiritual caregivers in implementing multidisciplinary spiritual care: a National Survey. Int J Environ Res Public Health.

[23] Ronaldson S (2012). Spirituality and spiritual caring: nurses’ perspectives and practice in palliative and acute care environments. J Clin Nurs.

[24] Sewkarran V, Gumede EZ (2023). Spiritual Care in action for Oncology patients in the uMgungundlovu and eThekwini Health districts, KwaZulu-Natal, South Africa: a qualitative study. SAGE Open Nurs.

[25] Egan R et al. New Zealand nurses’ perceptions of spirituality and spiritual care: qualitative findings from a National Survey. Religions 2017.

[26] Cooper KL et al. How nurses Understand spirituality and spiritual care: a critical synthesis. J Holist Nurs 2019. 10.1177/0898010119882153 31596165

[27] Hu Y, Tiew LH, Li F (2019). Psychometric properties of the Chinese version of the spiritual care-giving scale (C-SCGS) in nursing practice. BMC Med Res Methodol.

[28] Lukovsky J, McGrath E, Sun C, Frankl D, Beauchesne MA (2021). A survey of hospice and palliative care nurses’ and holistic nurses’ perceptions of spirituality and spiritual care. J Hospice Palliat Nurs.

[29] Tong A, Sainsbury P, Craig J. Consolidated criteria for reporting qualitative research (COREQ): a 32-item checklist for interviews and focus groups. INT J QUAL HEALTH C 2007; 19(6). 10.1093/intqhc/mzm042 17872937

[30] Clarke V, Braun V (2017). Thematic analysis. J Posit Psychol.

[31] Malik MM. Enhancing spiritual palliative care of Muslim patients: a perspective from Islamic theology. 2020.

[32] Hu Y, Jiao M, Li F (2019). Effectiveness of spiritual care training to enhance spiritual health and spiritual care competency among oncology nurses. BMC Palliat Care.

[33] Kaddourah B, Abu-Shaheen A, Al-Tannir M (2018). Nurses’ perceptions of spirituality and spiritual care at five tertiary care hospitals in Riyadh, Saudi Arabia: a cross-sectional study. Oman Med J.

[34] Farahani AS, Rassouli M, Salmani N, Mojen LK, Sajjadi M, Heidarzadeh M, Masoudifar Z, Khademi F (2019). Evaluation of health-care providers’ perception of spiritual care and the obstacles to its implementation. Asia-Pacific J Oncol Nurs.

[35] Rachel H, Chiara C, Robert K, Francesco S (2019). Spiritual care in nursing: an overview of the measures used to assess spiritual care provision and related factors amongst nurses. Acta Bio Medica: Atenei Parmensis.

[36] Juškauskienė E, Riklikienė O, Fisher J (2023). Spiritual well-being and related factors in Children with Cancer. J Pediatr Hematology/Oncology Nurs.

[37] Murgia C (2020). Spirituality in nursing: a concept analysis. Nurs Ethics.

[38] O’Brien M et al. Meeting patients’ spiritual needs during end-of‐life care: a qualitative study of nurses’ and healthcare professionals’ perceptions of spiritual care training. J Clin Nurs. 2018; 28. 10.1111/jocn.14648 30091251

[39] Mosavizadeh SR, Bahrami M, Maghami-Mehr A, Torkan M, Mehdipoorkorani L (2024). Explaining the nurses’ spiritual needs in the Oncology Department: a qualitative study. Iran J Nurs Midwifery Res.

[40] Khan NA, Siddiqui DA, Patience. Spirituality, suffering, and Life satisfaction: the mediatory role of patience in enabling Cognitive Flexibility, and emotional regulation. Available at SSRN; 2023.

[41] Evangelista C (2016). Spirituality in patient care under palliative care: a study with nurses. Esc Anna Nery.

[42] Marzband R, Hosseini S, Hamzehgardeshi Z. A Concept Analysis of Spiritual Care Based on Islamic Sources. *religions* 2016.

[43] Fortuna LR, Tobón AL, Anglero YL, Postlethwaite A, Porche MV, Rothe EM (2022). Focusing on racial, historical and intergenerational trauma, and resilience: a paradigm to better serving children and families. Child Adolesc Psychiatr Clin N Am.

[44] Stöckigt B (2021). Experiences and Perceived effects of Rosary praying: a qualitative study. J Relig Health.

[45] Suleman M (2023). The balancing of virtues—muslim perspectives on palliative and end of life care: empirical research analysing the perspectives of service users and providers. Bioethics.

[46] Jaberi A, Momennasab M, Yektatalab S, Ebadi A, Cheraghi MA (2019). Spiritual health: a concept analysis. J Relig Health.

[47] Laranjeira C, Dixe MA, Querido A (2023). Perceived barriers to providing spiritual care in Palliative Care among professionals: a Portuguese cross-sectional study. Int J Environ Res Public Health.

[48] Lee GE, Kim K (2020). Analysis of spiritual care experiences of acute-care hospital nurses. Korean J Hospice Palliat Care.

