# Supplementary material for: Exploring nurses’ experiences of providing spiritual care to cancer patients: a qualitative study
Source: BMC Nurs. 2024 Mar 27;23:207. doi: 10.1186/s12912-024-01830-2 (PMC10967064; doi:10.1186/s12912-024-01830-2)
Supplement: Supplementary file 1 — Supplementary Material 1 [file 12912_2024_1830_MOESM1_ESM.docx]

**Interview Guide**

1. Could you please share your thoughts on the concept of spirituality?
2. How do you define spirituality?
3. When you think of spiritual care, what comes to mind? What does spiritual care mean to you?
4. How do you perceive spiritual care within your responsibilities as an oncology nurse?
5. Tell me how you would recognize a patient’s spiritual care needs? You may like to give examples in practice.
6. Could you please list some examples of spiritual care?
7. Could you please share a time when you provided spiritual care and tell the story, including the details of when, how, and why?
8. In your view, how does the local cultural context influence your approach to spiritual nursing care?
9. Please share details of any relevant education, training, or work experience you have in the field of spiritual care. Additionally, please comment on how this background has prepared you to meet the spirituality needs in nursing care.
10. Please provide suggestions on how the organization and its key resources could assist you in delivering better spiritual care.
11. Based on your experience, what typically motivates you to deliver spiritual nursing care?
12. Based on your experience, what factors typically restrict or hinder you from delivering spiritual nursing care?
13. Could you please suggest anything that would facilitate spiritual nursing care?
14. Is there anything else you would like to say about spiritual care that we have not talked about?
